# Neutrophil percentage-to-albumin ratio is a new diagnostic marker for spontaneous bacterial peritonitis: a prospective multicenter study

**DOI:** 10.1186/s13099-024-00610-2

**Published:** 2024-04-01

**Authors:** Nasser Mousa, Mohamed salah, Sherif Elbaz, Alaa Elmetwalli, Amr Elhammady, Eman Abdelkader, Mostafa Abdelsalam, Niveen El-wakeel, Marwa Mansour, Manal Hashem, Ola El-Emam, Wesam Elderiny, Mohammed Abdelaziz, Ayman Elgamal, Alaa Habib

**Affiliations:** 1 Tropical Medicine Department, Faculty of Medicine, Mansoura University, Mansoura, Egypt; 2 Endemic Diseases and Gastroenterology Department, Aswan University, Aswan, Egypt; 3 Department of Clinical Trial Research Unit and Drug Discovery, Egyptian Liver Research Institute and Hospital (ELRIAH), Mansoura, Egypt; 4 Internal Medicine Department, Banha University, Benha, Egypt; 5 Internal Medicine Department, Mansoura University, Mansoura, Egypt; 6 Medical Microbiology and Immunology Department, Mansoura University, Mansoura, Egypt; 7 Department of Basic Medical Sciences, Faculty of Medicine, New Mansoura University, New Mansoura, Egypt; 8 Internal medicine department, Zagazig University, Zagazig, Egypt; 9 Clinical Pathology Department, Mansoura University, Mansoura city, Egypt; 10 Department of Tropical Medicine, Menoufia University, Menoufia, Egypt

**Keywords:** The neutrophil percentage-to-albumin ratio, Liver cirrhosis and spontaneous bacterial peritonitis

## Abstract

**Background:**

The neutrophil percentage-to-albumin ratio (NPAR) is a novel measure of systemic inflammation and infection. Low albumin levels increase the risk of infection, while high neutrophil counts indicate the presence of infection. Spontaneous bacterial peritonitis (SBP) is a serious infection in cirrhotic ascites, and the potential of NPAR in diagnosing SBP is not yet established.

**Objective:**

The objective of this study is to determine the diagnostic value of NPAR in identifying SBP.

**Patients:**

This prospective multicenter study included 465 patients diagnosed with cirrhotic ascites and SBP according to international guidelines. Demographic, clinical, and laboratory data were collected. The sensitivity and specificity of NPAR values for diagnosing SBP were assessed using the receiver operating characteristic curve.

**Results:**

For SBP diagnosis in the total cohort, NPAR of > 17 had a sensitivity of 85.71%, specificity of 66.67%, and 95% CI (42.1–99.6). In culture-positive SBP, the NPAR at a cut-off > 5.2 had a sensitivity of 85.71%, specificity of 83.33%, and 95% CI (0.709 to 0.979), while in culture-negative SBP, the NPAR at a cut-off > 2.1 had a sensitivity of 92.86%, specificity of 33.33% and CI (0.367 to 0.764). The multivariate analysis revealed that albumin (OR = 2.78, [1.11;3.98], INR (OR = 0.198, [0.066;0.596], creatinine (OR = 0.292, [0.1; 0.81], CRP (OR = 3.18, [1.239;4.52] total leukocytic count (TLC) (OR = 1.97, [1.878; 2.07], platelets (OR = 2.09, [0.99; 2.31] and neutrophil (OR = 3.43, [1.04;3.89] were significantly associated with higher prediction rates for culture positive SBP.

**Conclusions:**

NPAR could be a new, affordable, noninvasive test for diagnosing SBP.

## Introduction

Cirrhosis is a leading cause of death in many countries. Portal hypertension in the setting of liver cirrhosis is estimated to be the source of around 75% of cases of ascites, with the remaining instances arising from infectious, inflammatory, and infiltrative causes [1–6].

Cirrhotic patients are at increased risk for bacterial infections, which can lead to serious illness and death. One of the most severe infections in these patients is spontaneous bacterial peritonitis (SBP), which affects 10–30% of individuals [7–9]. The hospital mortality rate for spontaneous bacterial peritonitis is around 20%. However, improvements in identification and treatment may lead to a decline in the fatality rate [10, 11]. Neutrophils play a vital part in the innate cellular immune system. Previous research revealed a correlation between early, more significant neutrophil counts and enhanced sepsis severity. Counting peripheral leukocytes, such as neutrophils, is a simple and affordable method of identifying inflammation [12, 13].

Albumin is a highly soluble and stable negatively charged protein most abundant in human plasma. It plays important roles as a buffer, antioxidant, immunomodulator, antidote, and transporter in the plasma [14, 15].

Patients with advanced cirrhosis experience reduced albumin production and poor hepatocellular function, resulting in a 60–80% reduction. Albumin levels are a strong predictor of mortality in numerous studies of individuals with cirrhosis, making it an essential prognostic factor [16, 17]. The Child-Pugh-Turcotte score, commonly used to predict outcomes for cirrhosis, includes albumin as one of its components. Multiple studies have found a link between low albumin levels and poor clinical outcomes [15, 18].

A standard blood test can quickly obtain the neutrophil percentage-albumin ratio (NPAR). It has been identified as a predictor of prognosis in patients with various conditions, including malignancy, acute renal injury, septic shock, and cardiogenic shock [19–21].

To our knowledge, there has been no previous research on using NPAR as an indicator for diagnosing SBP. Therefore, this study aims to evaluate the clinical effectiveness of NPAR as a new, easy-to-use, affordable, and noninvasive biochemical test for diagnosing SBP.

## Patients and methods

In this prospective study, we aimed to enroll a total of 465 patients with liver cirrhosis and ascites referred to the Tropical and Internal Medicine Departments at Mansoura University, Benha University, and Zagazig University from October 2020 to June 2023 to achieve adequate statistical power for detecting a clinically meaningful difference in a new diagnostic marker for SBP. The sample size was determined based on a power of 80% and a significance level (alpha) of 0.05. We estimated the effect size based on a review of relevant literature, and input from clinical experts in the field was considered to refine our effect size estimate, resulting in a calculated sample size of 465 patients.

All participants underwent a comprehensive medical history, physical examination, and abdominal ultrasound. Triphasic CT scans were also performed as necessary.

### Exclusion criteria

Patients who were immunocompromised, had non-cirrhotic ascites (e.g., malignant or tubercular), had taken antibiotics before hospital admission, or were on prophylactic antibiotics for SBP or anticoagulants were excluded. Patients with heart or kidney failure, neoplastic or hematological illnesses, autoimmune disorders, secondary bacterial peritonitis from surgical causes, and associated infections (e.g., skin and lung infections) that could affect blood WBC levels were also excluded.

### Sampling

1. At the patient’s bedside, 15 ml of ascitic fluid samples were taken using the standard paracentesis technique under sterile settings. The sample was quickly added to the bedside vials for aerobic and anaerobic blood cultures (10 ml). Within three hours, the remaining ascitic fluid was subjected to biochemical and cytological investigation using tubes containing EDTA 30. According to international recommendations, spontaneous bacterial peritonitis (SBP) is diagnosed when the ascitic polymorphonuclear leukocytes (PMNL) cell count equals or exceeds 250/mm3 and the ascitic fluid culture is positive (referred to as culture-positive SBP) or when it is negative for bacterial growth but still contains neutrophils at a level higher than 250/mm3 (referred to as culture-negative neutrocytic ascites) and there are no other causes of peritonitis or hemorrhagic ascites [22, 23].
2. 5 ml of venous blood was taken during paracentesis. 2 ml was collected in a polystyrene EDTA tube for CBC analysis, precisely to measure neutrophil %. The remaining 3 ml were placed in a clotting tube for further testing. Centrifugation separated non-hemolyzed sera, which were used to test for creatinine and liver functions (ALT, AST, albumin, bilirubin, and prothrombin time). C-reactive protein from Roche Diagnostics was used to measure CRP levels using a particle-enhanced immunoturbidimetric technique. The neutrophil count was automatically determined using Abbott’s CELL-DYN Emerald cell counter in Wiesbaden, Germany.
3. The NPAR was calculated by dividing the percentage of neutrophils (the numerator) by the amount of albumin (the denominator) in the same blood samples obtained upon admission [24].

### Ethics approval

The Mansoura Faculty of Medicine Institutional Review Board **(MFM-IRB: R.23.07.2275)** approved the study, and all participants provided written permission before participating in any protocol-required procedure.

### Statistical analysis

Data was loaded and analyzed using IBM SPSS Statistics for Windows, version 22.0. Qualitative data was described using numbers and percentages. Quantitative data was assessed for normality using the Kolmogrov-Smirnov test and reported using median (minimum and maximum) for non-parametric data and mean and standard deviation for parametric data. Significance was determined at the 0.05 level. Chi-Square and Monte Carlo tests were used to compare groups, while the Student t-test and Mann-Whitney U tests were used to compare independent groups with normally and non-normally distributed data. A multivariable logistic regression analysis was employed to identify risk factors and predictors, with outcomes presented as odds ratios (OR) alongside 95% confidence intervals (CI). Receiver Operating Characteristic (ROC) curve analysis assessed diagnostic performance, revealing sensitivity and specificity. Adjusted odds ratios and 95% confidence intervals were computed.

## Results

**Fig. 1:**
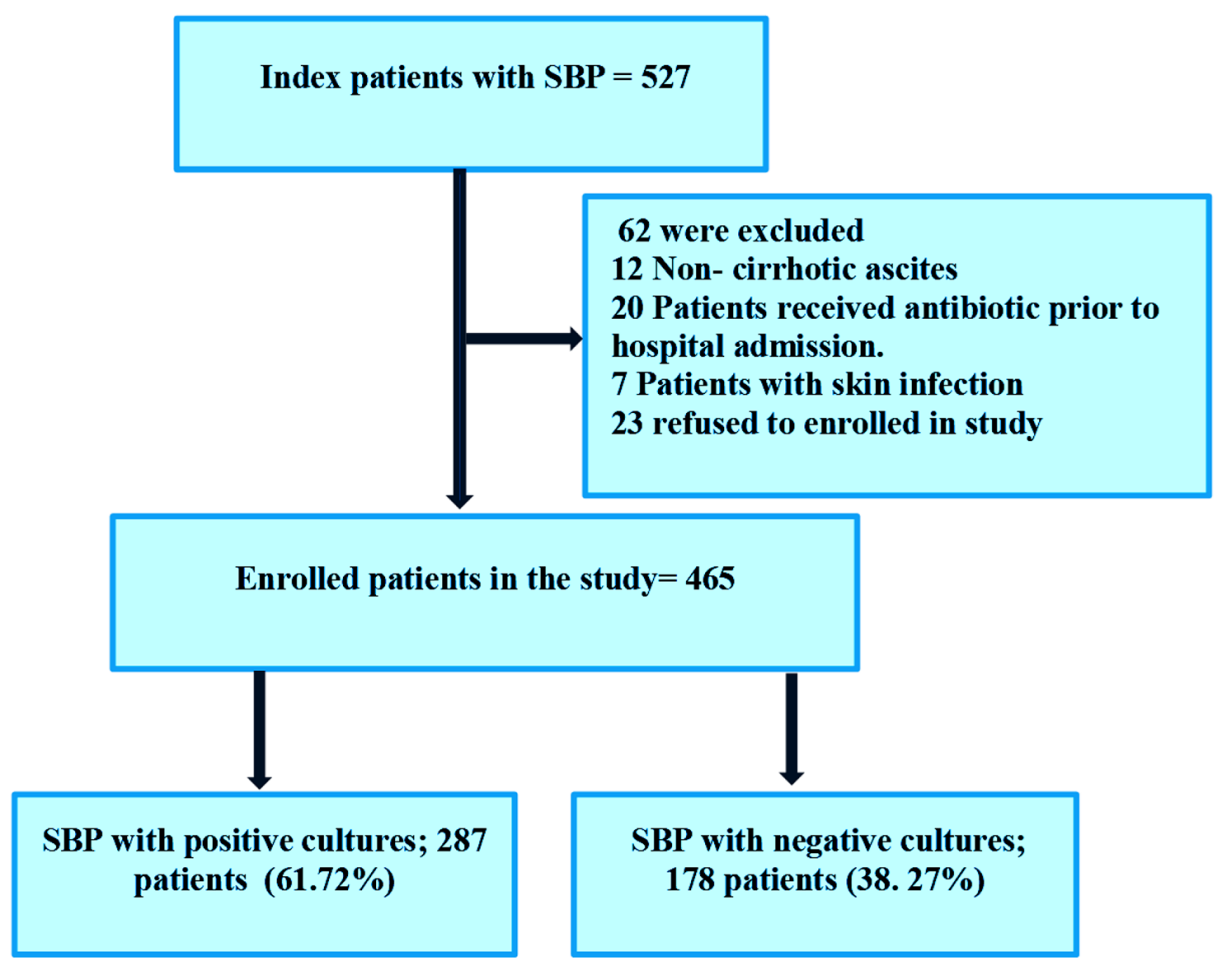
Flow chart of study patients

The study involved 527 patients with cirrhotic ascites and SBP. After excluding those who did not meet the criteria, 465 patients were included. Of these, 287 (61.72%) had culture-positive SBP, while 178 (38.27%) had culture-negative SBP as revealed in flow chart (Fig. 1).

Table 1 displays the demographics and clinical data of the groups studied. The two groups were similar in age and sex. Compared to cultures negative SBP, those with culture positive SBP showed a significant increase in the prevalence of HBV infection, fever, and abdominal pain and a notable decrease in HCV infection and jaundice occurrences. No significant differences were found in esophageal varices rupture, hepatic encephalopathy, and hepatorenal syndrome.

**Table 1:** Cohort characteristics based on demographics and clinical data

Characteristics	Total patients *N* = 465 (100%)	CPS *N* = 287 (61.72%)	CNS *N* = 178 (38.27%)	*P*-value
Age, (mean ± SD)	55.48 ± 1.35	56.60 ± 5.52	55.00 ± 8.42	0.123
**Gender, N (%)** - Male - Female	256 (55.05%) 209 (44.96%)	156 (54.35%) 131 (45.64%)	100 (56.17%) 78 (43.82%)	0.217
**Virology, N (%)** - HCV - HBV - Negative	369 (79.35%) 84 (18.06%) 12 (2.58%)	210 (73.17%) 69 (24.04%) 8 (2.78%)	159 (89.32%) 15 (8.42%) 4 (2.24%)	0.04 0.01 0.078
**Presentation, N (%)** - Fever - Abdominal pain - Jaundice - Similar attack - ROV - HE - RI	92 (19.78%) 103 (22.15%) 182 (39.13%) 14 (3.01%) 6 (1.29%) 55 (11.82%) 13 (2.79%)	61 (21.25%) 79 (27.52%) 96 (33.44%) 9 (3.13%) 4 (1.39%) 30 (10.45%) 8 (2.78%)	31 (17.41%) 24 (13.48%) 86 (48.31%) 5 (2.80%) 2 (1.12%) 25 (14.04%) 5 (2.80%)	0.022 0.004 0.047 0.127 0.141 0.231 0.523

**CPS**: culture-positive spontaneous bacterial peritonitis; **CNS**: culture-negative spontaneous bacterial peritonitis; **HCV**: hepatitis c virus; **HBV**: hepatitis B virus; **ROV**: rupture esophageal varices; **HE**: hepatic encephalopathy; **RI**: renal impairment

Table 2 displays the specific laboratory parameters of the study patients. Compared to cultures negative SBP, those with culture positive SBP showed a significant increase in ascitic PMNL and lymphocytes, as well as in the overall count of WBCs, neutrophils, lymphocytes, and platelets in the blood, while demonstrating a significant decline in hemoglobin levels. Regarding biochemistry, patients with cultures positive SBP showed a substantial increase in ALT, AST, bilirubin, and CRP and NPAR, while their serum albumin levels significantly decreased in contrast to those with cultures negative SBP. No significant differences were found regarding serum creatinine or INR.

According to multivariate analysis (Table 3). Our data revealed that albumin (OR = 2.78, [1.11;3.98], *p* = 0.0298), INR (OR = 0.198, [0.066;0.596], *p* = 0.0185), creatinine (OR = 0.292, [0.1; 0.81], *p* = 0.0185), CRP (OR = 3.18, [1.239;4.52], *p* = 0.008), total leukocytic count (TLC) (OR = 1.97, [1.878; 2.07], *p* = 0.041), platelets (OR = 2.09, [0.99; 2.31], *p* = 0.047] and neutrophil (OR = 3.43, [1.04;3.89], *p* = 0.0129) were associated with higher prediction rate for CPS. However, hemoglobin (OR = 1.15, [0.865;1.54], *p* = 0.333) and bilirubin (OR = 0.576, [0.13; 2.46], *p* = 0.4569), did not correlate with this prediction.

**Table 2:** Cohort-specific laboratory parameters

Characteristics	CPS *N* = 287 (61.72%)	CNS *N* = 178 (38.27%)	*P*-value
**Ascitic fluid, (mean ± SD)** - WBC - PMNLs - Lymphocytes	6404 ± 295 71.64 ± 12.02 25.56 ± 15.07	3492 ± 568 57.17 ± 1.70 16.14 ± 1.73	0.001 0.012 0.049
**CBC** - Hgb (g/dL) - WBCs (× 10^3^/µL) - Neutrophils (%) - Lymphocytes (× 10^3^/µL) - Platelets (× 10^3^/µL)	10.26 ± 0.20 10.70 ± 2.23 74.00 ± 8.86 23.02 ± 2.12 113.48 ± 9.12	11.43 ± 0.95 7.82 ± 1.62 58.19 ± 8.26 15.38 ± 4.25 81.98 ± 4.66	0.042 0.021 0.001 0.029 0.041
**Biochemical characteristic** - ALT (U/L) - AST (U/L) - Albumin (g/dL) - Bilirubin (mg/dL) - Creatinine (mg/dL) - INR (%) - CRP (mg/dL) - NPAR	42.00 ± 4.12 81.00 ± 5.12 2.10 ± 0.21 5.20 ± 1.21 2.60 ± 0.25 1.80 ± 0.12 73.00 ± 7.6 35.24 ± 2.31	28.00 ± 3.4 61.00 ± 5.2 2.90 ± 0.10 3.40 ± 0.77 1.65 ± 0.27 1.60 ± 0.08 46.00 ± 5.1 20.07 ± 1.98	0.044 0.032 0.010 0.002 0.090 0.240 0.026 0.001

**Abbreviation: CPS**: culture-positive spontaneous bacterial peritonitis; **CNS**: culture-negative spontaneous bacterial peritonitis; **CBC**: complete blood count; **WBC**: white blood cells, **PMNLs**: polymorph nuclear leukocytes, **Hgb**: hemoglobin, **ALT**: alanine transferase, **AST**: aspartate transferase, **INR**: international normalized ratio, **CRP**; C-reactive protein; **NPAR**: neutrophil percentage-albumin ratio. Data were expressed as mean ± SD

**Table 3:** An analysis of multivariate data using logistic regression to predict SBP

Variable	Odds ratio (OR)	95% confidence interval (CI)	*P*-value
**Albumin(g/dL)**	2.78	[1.11;3.98]	0.029^*^
**Bilirubin (mg/dL)**	0.576	[0.135;2.46]	0.457
**INR**	0.198	[0.066;0.596]	0.004^**^
**Creatinine (mg/dL)**	0.292	[0.105;0.813]	0.018^*^
**CRP (mg/dL)**	3.18	[1.239;4.52]	0.008**
**TLC (× 103/µL)**	1.97	[1.878;2.07]	0.041^*^
**Platelets (× 103/µL)**	2.09	[0.994;2.31]	0.047^*^
**Neutrophil (%)**	3.43	[1.04;3.89]	0.012^*^
**HB (g/dL)**	1.15	[0.865;1.54]	0.333

**Abbreviation: TLC**: total leukocytic count; **HB**: hemoglobin; **INR**: international normalized ratio, **CRP**; C-reactive protein. * *p* < 0.05, **p < 0.01

**Fig. 2:**
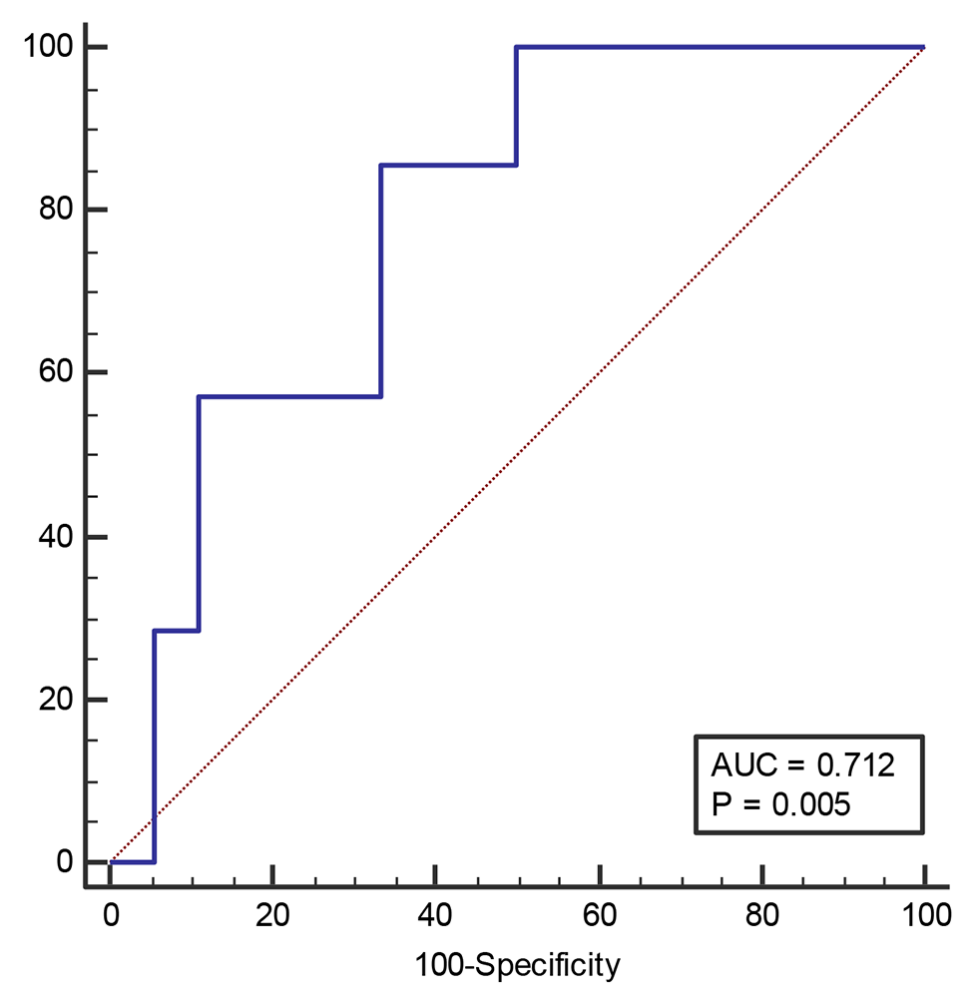
Receiver operator characteristics curve for NPAR in total SBP

Table 4 reveals the total SBP area under the ROC curve (AUC). In our study, we found a significant AUC of 0.71 when the cut-off was set at > 17 (Fig. 2). The sensitivity was found to be 85.71%, with a 95% confidence interval (CI) of 42.1 to 99.6, while the specificity was 66.67%, with a 95% CI of 41.0 to 86.7. These results highlight the usefulness of this method for predicting SBP in patients. The -LR (likelihood ratio) of 2.57 and the Youden Index of 0.21 further support the effectiveness of this approach and encourage us to explore it further.

**Table 4:** Area under the ROC curve (AUC) in total SBP

Cut-off	AUC	Sensitivity	95% CI	Specificity	95% CI	+LR	-LR	Youden Index
> 17	0.71	85.71	42.1–99.6	66.67	41.0–86.7	2.57	0.21	0.524

**Fig. 3:**
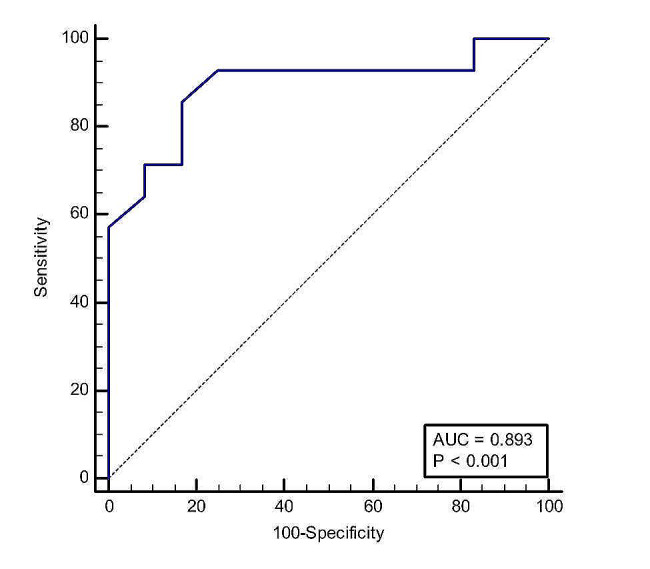
Receiver operator characteristics curve for NPAR in culture-positive SBP

**Fig. 4:**
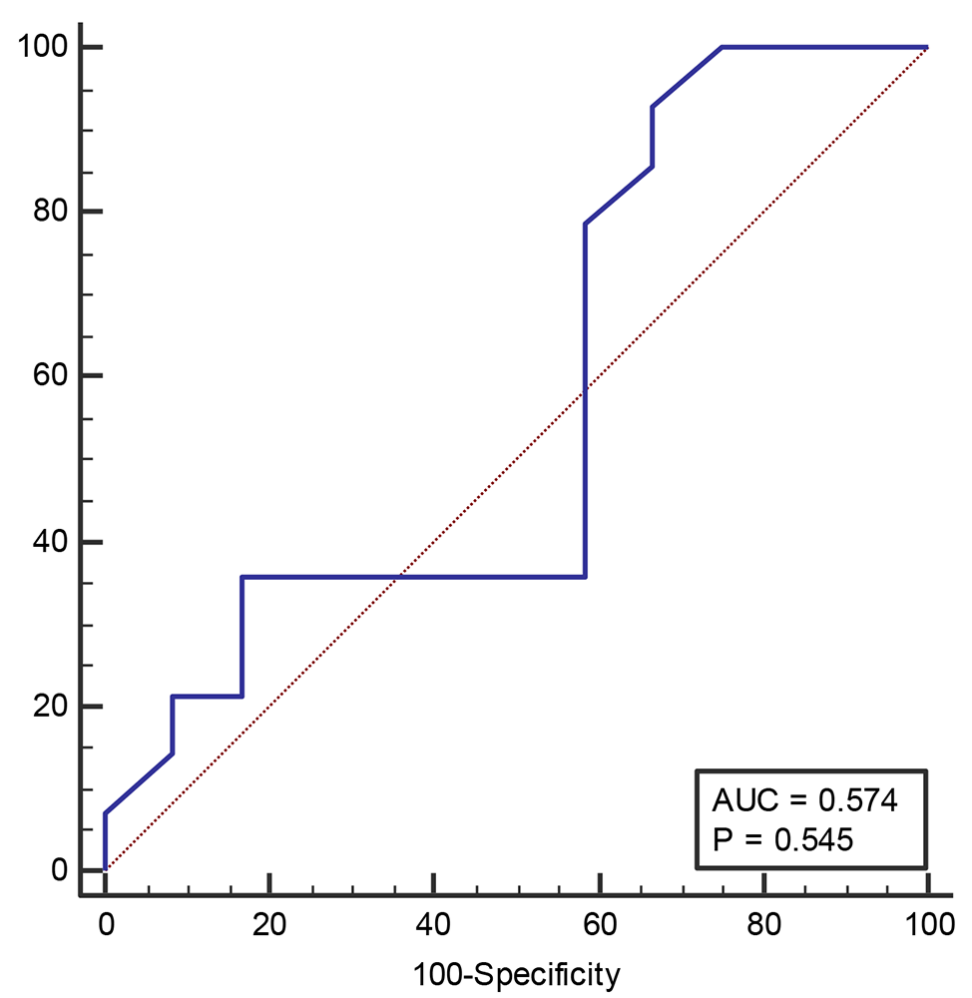
Receiver operator characteristics curves for NPAR in culture-negative SBP

Furthermore, Table 5 shows the Area Under the ROC Curve (AUC) in culture-positive SBP. The data reveals the AUC, sensitivity, specificity, -LR, and Youden Index for different cut-off values. For instance, setting the cut-off at > 5.2, the AUC was found to be 0.893, which is significant (Fig. 3). The sensitivity was 87.85%, with a 95% CI of 57.2 to 98.2, while the specificity was 83.33%, with a 95% CI of 51.6 to 97.9. The -LR was 5.14, and the Youden Index was 0.71. These results suggest that this method is effective in predicting culture-positive SBP in patients. Additionally, an NPAR at a cut-off > 2.1 functioned optimally in culture-negative SBP, with a sensitivity of 92.86% and a specificity of 33.33% (Table 6 and Fig. 4).

**Table 5:** Area under the ROC curve (AUC) in culture-positive SBP

Cut-off	AUC	Sensitivity	95% CI	Specificity	95% CI	+LR	-LR	Youden Index
> 5.2	0.893	87.85	57.2–98.2	83.33	51.6–97.9	5.14	0.71	0.783

**Table 6:** Area under the ROC curve (AUC) in culture-negative SBP

Cut-off	AUC	Sensitivity	95% CI	Specificity	95% CI	+LR	-LR	Youden Index
> 2.1	0.574	92.86	66.1–99.8	33.33	9.9–65.1	1.39	0.21	0.324

## Discussion

Spontaneous bacterial peritonitis in cirrhotic patients worsens their prognosis, leading to liver decompensation, sepsis, and multi-organ failure. It also increases mortality rates and causes a 25.6% re-hospitalization rate within 30 days, resulting in significant medical, financial, and emotional burden on patients [23, 25]. According to global guidelines, the primary method for diagnosing SBP is through diagnostic paracentesis [23]. However, this procedure is invasive and carries risks. A new inflammation-based predictor, the neutrophil-albumin ratio (NPAR), has been discovered. The NPAR uses routine blood tests to obtain neutrophil and albumin values. Research on the diagnostic usefulness of the NPAR ratio for SBP is limited.

Our study found that an NPAR of > 17 effectively predicts SBP diagnosis with a sensitivity of 85.71% and specificity of 66.67%. The narrow 95% CI range indicates a reliable model for SBP diagnosis. In culture-positive SBP, an NPAR value above 5.2 can accurately detect 85.71% of cases with a sensitivity of 85.71% and specificity of 83.33%. In culture-negative SBP, an NPAR value above 2.1 has a sensitivity of 92.86% and a specificity of 33.33%. The 95% confidence interval indicates high reliability in our results.

Previous studies have examined the significance of neutrophils [26, 27] and serum albumin [28, 29] as prognostic indicators for identifying individuals with SBP. However, to our knowledge, no prior research has focused on the ratio of neutrophil percentage to albumin as an indicator of SBP.

As per our findings, many studies have confirmed the significance of neutrophils as an indicator of infections [30]. Analyzing the level of neutrophils in the blood is a cost-effective and easily accessible method for identifying bacterial infections. Russell et al. showed that the ratio of leukocytes in the blood is a valuable biomarker for detecting infection [31]. Neutrophils are the most common type of white blood cells and play a crucial role in defending against microbial invasion [20]. Cirrhotic patients may have low neutrophil levels due to an enlarged spleen. A high white blood cell count could indicate a microbial infection, while a rising neutrophil count may suggest that the infection has not been fully cleared [32]. This resistance to infection and incomplete clearance can lead to increased production of neutrophils by the bone marrow [30]. Neutrophils protect cirrhotic patients from infection, performing activities like phagocytosis and releasing reactive oxygen species. Cirrhotic ascites increases intestinal permeability, raising the risk of bacterial translocation and SBP [33].

Kasztelan-Szczerbinska et al. supported our results by showing that an increase in leukocyte count in peripheral blood can indicate the development of SBP in individuals with ascites [34]. Jiang et al. also found that patients with SBP have a higher PMN count compared to those without SBP. Additionally, a strong positive correlation between ascitic neutrophil count and serum leukocyte count was established [35].

Albumin is the second component of NPAR and is linked to the development and severity of bacterial infections. It can also predict infectious complications in non-infective diseases [36]. Low serum albumin levels are a marker for infection because they can directly affect the body’s ability to fight infections. The relationship between serum albumin levels and the onset and severity of infectious diseases may be due to the effects of inflammation on albumin levels [37]. Albumin has been shown to predict SBP in multiple studies [28, 29]. It also plays a valuable role in preventing SBP and reducing its complications [30]. This may be due to albumin’s ability to bind vasodilators such as NO, IL-6, and TNF-a, leading to lower concentrations of these inflammatory markers in the plasma and ascitic fluid after albumin infusion [38]. Our findings suggest using NPAR as a diagnostic marker for SBP, as previous studies have also found it to be more sensitive than conventional markers in predicting poor infection outcomes [24].

One of the limitations of this study was the interpretation of the ROC curve within the context of our study design. We acknowledge the fundamental limitation posed by the absence of true negatives (TN) and false positives (FP) in our study population, which precludes the calculation and interpretation of specificity in the traditional sense. Given this limitation, we recognize the importance of exploring alternative approaches for evaluating the test’s diagnostic performance and interpreting the ROC curve analysis findings within the context of our study population. The alternative approaches were firstly focused on sensitivity and positive and negative likelihood ratio (LR); given that all participants in our study are positive for SBP, sensitivity (the ability of the test to identify individuals with the condition correctly) and + LR (the probability that individuals with a positive test result genuinely have the condition) are more relevant measures for evaluating the diagnostic performance of the test.

Secondly, consider clinical utility; beyond statistical measures, it’s essential to assess the test’s clinical utility in our study population. This included evaluating its ability to detect SBP in clinically relevant scenarios accurately and its impact on patient management and outcomes. Thirdly, examine external validity; while our study population may not reflect the broader patient population, it’s crucial to consider the external validity of the test’s performance in similar clinical settings or patient cohorts.

## Conclusions

NPAR may create a more accurate and reliable biomarker for predicting SBP. NPAR has the advantage of being a simple and potentially novel biomarker that can be quickly and easily obtained from admission laboratory data.

## Data Availability

The data used in this study is available upon a reasonable request to the corresponding author and after permission from all participating services.
